# A role for complement blockade in kidney transplantation

**DOI:** 10.1038/s41423-022-00854-5

**Published:** 2022-03-24

**Authors:** Diana Karpman, Zivile Bekassy, Anne Grunenwald, Lubka T. Roumenina

**Affiliations:** 1 Department of Pediatrics, Clinical Sciences Lund, Lund University, Lund, Sweden; 2 Centre de Recherche des Cordeliers, INSERM, Sorbonne Université, Université de Paris, F-75006 Paris, France

**Keywords:** Complement cascade, Biomarkers

Kidney transplantation is a life-saving procedure for patients with end-stage renal failure, but unfortunately, only a limited number of patients benefit from this procedure due to organ shortage. Moreover, even after transplantation, the graft can be lost due to antibody-mediated rejection (AMR). A recent study by Schmitz et al. in *Nature Communications* [1] provides new hope to prevent AMR and prolong renal allograft survival. The authors inhibited the innate immune complement cascade at the level of its central component C3 and successfully prevented the tissue injury mediated by donor-specific antibodies (DSAs) in a primate model.

After kidney transplantation, graft function can be impaired by various injurious processes. HLA incompatibility between the donor and the recipient contributes to the development of AMR. Events preceding organ removal from the donor, being living-related, living-unrelated, or deceased, and the length of ischemia followed by reperfusion can affect the immediate outcome [2]. Additionally, immunosuppressive drugs targeting T-cell-mediated rejection may cause renal toxicity at higher concentrations, and conversely, poor compliance with immunosuppressive medication may affect graft function. Viral infections, such as cytomegalovirus (CMV) or BK virus infections, as well as a recurrence of the primary kidney disease, may also cause graft dysfunction.

Complement activation in the graft may occur during ischemia, resulting in extensive endothelial cell damage. Prolonged ischemia also leads to complement C3 production by tubular cells, which correlates with postischemic graft failure in a mouse model [3]. The role of C3, central to all three complement pathways, and the C3 convertase, in the damage induced by ischemia/reperfusion was further demonstrated in mice using an antibody to Factor B that prevented tubular C3b deposition and systemic release of C3a [4]. In addition to ischemia/reperfusion, complement activation in a kidney graft may be the result of the recurrence of the primary disease, if complement-mediated or complement-driven, such as C3 glomerulopathy, atypical hemolytic uremic syndrome, IgA nephropathy or vasculitis, as well as other vascular diseases. In addition, prior and repeated exposure of the recipient’s plasma to hemodialysis biofilters before transplantation can activate the complement system in the bloodstream [2].

One of the most common causes of graft deterioration is AMR. AMR is associated with the presence of DSAs in the circulation of sensitized graft recipients [5]. These antibodies are directed to HLA antigens present on donor cells. DSAs induce complement-dependent cytotoxicity, initiated by binding C1q or C3d, as well as complement-independent cytotoxicity (as reviewed previously [5]). AMR is usually treated by plasma exchange, protein A immunoadsorption, anti-CD20 antibodies to deplete B cells, immunoglobulin infusions, and in certain cases with bortezomib, antithymocyte globulin (ATG), and corticosteroids [5]. Although patients can be treated to decrease DSA levels before transplantation, an increase in DSAs posttransplantation can promote rejection [6].

The role of complement activation, specifically of C3, in AMR was highlighted in the study by Schmitz et al., in which a C3 inhibitory peptide, Cp40, was administered to HLA-sensitized primates to prevent AMR after kidney transplantation [1]. Sensitization of the recipient primates was achieved by repeated skin transplants from the donor. This was followed by kidney transplantation from the same rhesus macaque donor. Altogether, eleven primates underwent the procedure, of which six were treated with Cp40 and five were controls. Primates were treated with Cp40 for 16 days, from 2 days before transplantation until Day 14 when treatment was discontinued. Primates also received conventional immunosuppressive therapy for solid organ transplantation, including tacrolimus, mycophenolate mofetil, methylprednisolone, rhATG and CMV prophylaxis. Cp40 treatment significantly improved renal function and prolonged graft survival. In addition to its expected direct effect on complement activation, Cp40 modulated T- and B-cell activation and proliferation, presumably by reduced signaling via C3aR and C5aR1 [1]. In Cp40-treated primates, 3/6 animals developed early rejection, which was associated with the presence of IgM DSAs, and a subgroup of animals exhibited prolonged graft survival and accommodation despite circulating DSAs. Ultimately, all treated animals developed AMR, and the authors concluded that combining C3 inhibition with antibody reduction may be more effective.

This is a very interesting proof-of-concept paper showing that C3 inhibition has a place in the treatment of AMR. The authors suggest that increasing the dose of Cp40 may be able to overcome the effects of IgM DSAs and prevent early rejection.

**Fig. 1:**
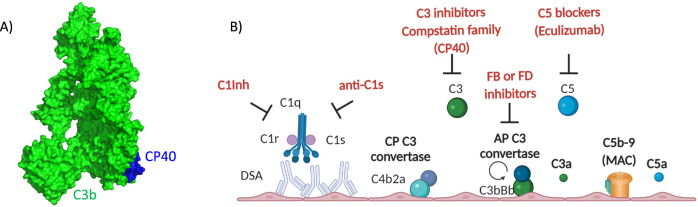
Complement blockade in antibody-mediated rejection. **A** Structural representation of the compstatin family C3 inhibitor CP40 (blue) bound to C3b (green), PDB ID 7BAG, generated using PyMol. **B** Simplified presentation of the complement cascade showing classical pathway activation by donor-specific antibodies (DSAs) and the amplification loop of the alternative pathway. C1 inhibitor (C1Inh) and anti-C1s block the classical pathway, Factor B (FB) and Factor D (FD) inhibitors block the alternative pathway, and C5 inhibitors (such as eculizumab) block the terminal pathway, while the compstatin family inhibitors of C3, such as CP40 used by Schmitz et al., block the central step of the cascade and hence all pathways. Generated using Biorender.com. CP classical pathway, AP alternative pathway, MAC membrane attack complex

CP40 is a peptidic C3 inhibitor of the compstatin family [7]. Compstatins bind to a shallow pocket on the surface of C3, formed by the macroglobulin ring of the β chain of human and primate C3 [8], and selectively block convertase-mediated cleavage of C3 regardless of the initiation pathway (Fig. 1A). C3 blockade has the advantage of shutting down all complement pathways, preventing the release of the anaphylatoxins C3a and C5a, C3b cell opsonization, and the formation of the membrane attack complex, all of which can contribute to tissue injury during AMR. This is a major advantage compared to other possible complement-inhibiting strategies (Fig. 1B). On the downside, as no complement pathway will be left functional, there is a risk of infections, which must be dealt with by vaccination and/or antibiotic prophylaxis, and potentially long-term immune complex and debris accumulation, as their clearance is partially dependent on C3b opsonization.

Other complement blocking strategies are also currently available in clinical practice. Inhibition of the terminal complement pathway by the C5 antibody eculizumab has been used for the treatment of AMR in kidney transplantation with some success in patients with evidence for complement-dependent AMR [9]; however, upstream complement activation at the level of C3 and C4 still occurred, as reviewed [2]. C5 blockade has the advantage of leaving the C3b-opsonization capacity intact for defense against pathogens and the clearance of immune complexes and cell debris but also allows excessive production of C3a possessing inflammatory properties. C1 inhibitors [10] (clinically available) and C1s antibodies [11] are other options, as they block the classical pathway triggered by DSAs. Indeed, in the study of Schmitz et al. [1], IgM conferred resistance to Cp40 treatment, underscoring the importance of the classical pathway. Blocking the classical pathway leaves the amplification loop of the alternative pathway intact, which has an advantage in pathogen defense, but the drawback is that it may not fully prevent ischemia/reperfusion-induced alternative pathway overactivation. A phase III clinical trial of C1 inhibitor treatment in AMR was prematurely terminated due to futility (NCT02547220), but a phase II trial using a monoclonal antibody targeting C1s is currently ongoing (NCT05156710). Finally, Factor B and Factor D inhibitors may also be of interest, as they will inhibit the alternative pathway, but they will leave the classical pathway, the main culprit, unattended.

A clinical trial of C3 inhibition in AMR will show whether this treatment strategy provides new hope for patients with kidney graft rejection. The transplantation field has the unmet need to find the right complement inhibitor and the appropriate combination therapy to prevent rejection not only of kidney allografts but also of other organs and even xenografts, as we have recently witnessed the first pig-to-human heart transplantation.
